# Associations between stressors and difficulty sleeping in critically ill patients admitted to the intensive care unit: a cohort study

**DOI:** 10.1186/s12913-020-05497-8

**Published:** 2020-07-09

**Authors:** Roberto Carlos Miranda-Ackerman, Mariana Lira-Trujillo, Alma Carolina Gollaz-Cervantez, Ana Olivia Cortés-Flores, Carlos José Zuloaga-Fernández del Valle, Luis Alberto García-González, Gilberto Morgan-Villela, Francisco José Barbosa-Camacho, Kevin Josue Pintor-Belmontes, Bertha Georgina Guzmán-Ramírez, Aldo Bernal-Hernández, Clotilde Fuentes-Orozco, Alejandro González-Ojeda

**Affiliations:** 1 Hospital San Javier, Guadalajara, Jalisco Mexico; 2 Unidad de Investigación Biomédica 02, Hospital de Especialidades del Centro Médico Nacional de Occidente, Instituto Mexicano del Seguro Social, Avenida Belisario Domínguez # 1000 Col. Independencia, 44340 Guadalajara, Jalisco Mexico

**Keywords:** Critically ill patient, ICU, Sleep, Stressors

## Abstract

**Background:**

Patients admitted to the intensive care unit (ICU) experience sleep disruption caused by a variety of conditions, such as staff activities, alarms on monitors, and overall noise. In this study, we explored the relationship between noise and other factors associated with poor sleep quality in patients.

**Methods:**

This was a prospective cohort study. We used the Richards–Campbell Sleep Questionnaire to explore sleep quality in a sample of patients admitted to the ICU of a private hospital. We measured the noise levels within each ICU three times a day. After each night during their ICU stay, patients were asked to complete a survey about sleep disturbances. These disturbances were classified as biological (such as anxiety or pain) and environmental factors (such as lighting and ICU noise).

**Results:**

We interviewed 71 patients; 62% were men (mean age 54.46 years) and the mean length of stay was 8 days. Biological factors affected 36% and environmental factors affected 20% of the patients. The most common biological factor was anxiety symptoms, which affected 28% of the patients, and the most common environmental factor was noise, which affected 32.4%. The overall mean recorded noise level was 62.45 dB. Based on the patients’ responses, the environmental factors had a larger effect on patients’ sleep quality than biological factors. Patients who stayed more than 5 days reported less sleep disturbance. Patients younger than 55 years were more affected by environmental and biological factors than were those older than 55 years.

**Conclusions:**

Patient quality of sleep in the ICU is associated with environmental factors such as noise and artificial lighting, as well as biological factors related to anxiety and pain. The noise level in the ICU is twice that recommended by international guides. Given the stronger influence of environmental factors, the use of earplugs or sleeping masks is recommended. The longer the hospital stay, the less these factors seem to affect patients’ sleep quality.

## Background

Sleep is a basic human need and is closely related to health and disease recovery. From the moment they enter an intensive care unit (ICU), patients in a critical state must deal with a variety of challenges that require adaptation to the ICU environment [1, 2].⁠ This experience can be unfamiliar and disorienting, and patients’ interactions and sensations can be limited. For example, ICU patients are subjected to continuous stimuli that can alter their sleep schedule [3, 4].⁠ The main sources of disturbance of ICU patients’ sleep include nurse and physician activities, family visits, and alarms [5–7].⁠ Patients in the ICU may remain in a chronic state of alertness because of the continuous sounds of alarms. Ryherd et al. exposed volunteers to an environment that simulated the ICU and found sleep alterations and elevated levels of biochemical markers of stress [8]⁠.

Poor sleep quality adversely affects vegetative functions and peripheral vascular tone, both of which are closely related to the recovery process in critical patients [9]⁠. Poor sleep quality can alter the immune response, which may increase the patient’s susceptibility to infection. Studies report that sleep disturbance can also cause disequilibrium between the sympathetic and parasympathetic nervous systems, which can change arterial blood pressure, cause tachycardia, increase oxygen consumption, induce hypoxemia, and decrease erythropoiesis [10, 11]. This is particularly important for ICU patients because their clinical evolution depends on both the etiology and treatment options available. ICU patients’ recovery can be impaired by preexisting sleeping disorders, which when added to those caused by the ICU environment, may worsen their experience in the ICU [12].

Studies have reported a relationship between the ICU environment and the perception of interrupted sleep or difficulties falling asleep. Noise is generated by a variety of sources within the ICU, such as conversations between nursing and medical staff, opening and closing of doors, family visits, ringing telephones, and noise from radios, televisions, and patient monitoring systems [5, 13, 14]. In addition, the placement of equipment can limit the inability to move freely, which can contribute to patient discomfort [2, 15]. The World Health Organization (WHO) suggests that noise levels inside a hospital should be 35 dB (dB) during the day and 30 dB at nighttime, although these requirements are not always met [16]⁠. ICU staff need to be aware of how environmental and biological factors can affect ICU patients’ sleeping patterns and their ability to rest.

## Methods

### Aims

The aims of this study were to identify factors associated with difficulty sleeping in critically ill patients in the ICU of a private hospital and to compare the effects of biological and environmental factors, such as noise level within the ICU, on patients’ sleep. The study’s hypothesis was that environmental and biological factors have detrimental effects on patients’ sleep quality in the ICU.

### Design

This was a prospective cohort study that evaluated the influence of biological and environmental factors in critically ill patients admitted to the ICU in a private hospital. The ICU is divided into a general ICU (8 beds) and a step-down unit (8 beds), representing a total of 16 beds. The step-down unit provides an intermediate level of care for patients with higher requirements than can be delivered in the general ward. The participant inclusion period was from January 2019 to May 2019.

The research team conducted interviews of all patients admitted to the ICU. These interviews took place during the morning shift each day for the length of the participants’ ICU stay. The procedures used for data collection are described in the following paragraphs.

To measure sleep quality, we used the validated Spanish language version of the Richards–Campbell Sleep Questionnaire (RCSQ) [17]. For this questionnaire, the patient is given a 100 mm visual analogue scale and places a mark on the line that best represents the quality and quantity of sleep. The line has two options at each extreme: one to record the best quality (“deep sleep”) and on other the worst quality (“light sleep”). To obtain a final score, the patient’s answers are measured with a ruler. A total score of 0–33 represents poor quality sleep, 34–66 represents average quality sleep, and 67–100 represents good quality sleep.

Afterwards, we asked the patients to give a reason why their sleep was disturbed. We categorized the stressors into factors as referred to in the North American Nursing Diagnosis Association (NANDA) [18], i.e., environmental factors and biological factors. Environmental factors included noise, temperature, and lighting in the ICU. Biological factors included pain, dyspnea, delirium, vomiting, encephalopathy, bronchospasm, diarrhea, and anxiety. Anxiety was defined as an anxious adjustment disorder in patients who mentioned feeling anxious to the ICU physicians, nurses, or medical staff. The following symptoms were assumed to relate to signs of patient anxiety: feeling nervous, irritable, and/or afraid because of their condition or treatment/procedures, fear of losing control, and somatic symptoms such as tachycardia, sweating, restlessness, and trembling) [19]. These stressors were measured using a survey that included questions about the causes of the patients’ sleep disturbance.

Additionally, we measured the noise levels within each of the ICUs. The sound data were collected using a microphone with a sensitivity range of 0–100 dB. Sound was measured three times a day in the morning, afternoon, and night on each day. The sound was recorded in occupied rooms for 60 min from the middle of the unit to obtain a general idea of the sound panorama inside of the unit for each shift. We included these data in the database for the corresponding shift (morning, afternoon, or night shift). Finally, we averaged the sound levels obtained for each patient to obtain the mean sound levels for each unit. The sound levels were recorded by an ICU physician, nurse, or medical student.

### Sample and participants

#### Sample size

The sample size was calculated according to the prevalence of sleep difficulties in patients hospitalized in general wards (50%) compared with those patients admitted to the ICU (80%) [20].⁠ We used a difference between proportion formula, a two-sided confidence level of 95%, and a power of 80% to calculate a minimum sample size of 66 patients. The total number of patients admitted to the ICU during the recruitment time was 157. Our final sample included 71 patients who met the following inclusion criteria.

##### Inclusion criteria

1. Age > 18 years
2. Patients admitted in the ICU or step-down unit for > 24 h
3. Patients who were not prescribed any adjuvant sleeping medication (such as benzodiazepines, dexmedetomidine, or haloperidol)
4. Patients with no preexisting sleep disorders (such as insomnia, obstructive sleep apnea, circadian rhythm sleep syndrome, or narcolepsy).

During the admission, patients were asked whether they took any medication (including adjuvant sleep medication) or if they had any chronic condition (the admission form includes an item for mental disorders).

##### Exclusion criteria

1. Patients prescribed an adjuvant sleeping medication during their ICU stay
2. Patients prescribed adjuvant sleeping medications before admission to the ICU
3. Patients with a preexisting sleeping disorder
4. Patients with a preexisting anxiety disorder.

The patients were divided into age groups, and the ICU length of stay was divided into groups using the median split. For the age groups, 36 patients (50.7%) were included in the ≤55-year-old group and 35 patients (49.3%) in the > 55-year-old group. For ICU stay length, 38 patients (53.5%) were included in the ≤5 days group and 33 patients (46.5%) were included in the > 5 days group.

### Data analysis

Data were analyzed using Statistical Package for Social Sciences (version 23 for Windows; IBM SPSS, Armonk, NY, USA). Descriptive statistics included percentage, mean, and standard deviation. Inferential analysis was performed using the chi-square test or Fisher’s exact test. Levene’s test was used to test for equality of variances for quantitative variables and showed that the data had a normal distribution. Student’s *t* test and analysis of variance (ANOVA) were used to analyze continuous variables. A two-sided *p* < 0.05 was considered to be significant.

## Results

We interviewed 71 patients during their hospital stay: 44 (62%) men and 27 (38%) women. Their mean age was 54.46 ± 18.4 years (range 16–92 years).

We examined whether the patients had a full night of sleep according to the length of hospital stay. In the group of patients who stayed ≤5 days in the ICU, 38 (81.5%) reported disturbed sleep, and seven patients (18.4%) had a full night of sleep during their stay. Of the 33 patients who stayed > 5 days in the ICU, only one reported having a full night of sleep, and 32 patients (96.9%) reported disturbed sleep. However, the differences between groups did not reach statistical significance (*p* = 0.060).

In the ≤55-year-old group, two patients reported they had slept all night without disturbances, and 34 patients (94.4%) reported disturbed sleep. In the > 55-year-old group, six patients (17.1%) reported a full night of sleep, and 29 patients (82.8%) reported disturbed sleep. The differences were not significant (*p* = 0.151).

### Stressors

**Fig. 1:**
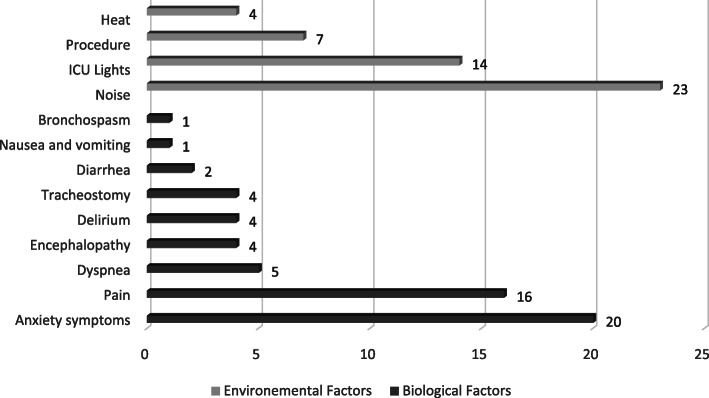
Prevalence of biological and environmental stressors

The most common environmental factor was noise, which was reported by 23 patients (32.4%), followed by ICU lighting (14 patients, 19.70%). When asked about which source of the sound patients found more disturbing, the primary sources were staff activities, telephones ringing, monitors and infusion pump alarms, and chairs moving. The most common biological factor was anxiety, which was reported by 20 patients (28.20%), followed by pain (16 patients, 22.5%). A complete list of stressors and their frequency is shown in Fig. 1.

When asked daily during their ICU stay if they had slept at night, 63 patients (88.7%) mentioned disturbed sleep during their ICU stay, and eight patients (11.3%) said they had slept without disturbance on all nights. The mean RCSQ score was 59.66 ± 15.18. The RCSQ scores according to stressors, age, and ICU stay length are shown in Table 1.

**Table 1:** RCSQ scores according to age, ICU stay length, and stressors

	RCSQ score	***p*** value
**Age groups**		
≤ 55 years	57.17 ± 13.69	0.163
> 55 years	62.22 ± 16.39	
**Duration of ICU stay**		
≤ 5 days	59.99 ± 17.41	0.848
> 5 days	59.29 ± 12.40	
**Stressors**		
***Biological factors***		
Anxiety symptoms		
Yes	60.70 ± 11.54	0.701
No	59.22 ± 16.54	
Pain		
Yes	57.53 ± 11.62	0.527
No	60.28 ± 16.11	
***Environmental factors***		
Noise		
Yes	49.26 ± 14.20	0.001
No	64.65 ± 13.06	
Lighting		
Yes	49.06 ± 16.54	0.003
No	62.27 ± 13.77	

*RCSQ* Richards–Campbell Sleep Questionnaire

The data are expressed as mean ± standard deviation, *p* values were obtained using Student’s t test

Analysis of the most frequent factors mentioned by patients (anxiety, pain, noise, and lights) showed that the most frequent factors associated with disturbed sleep at night were the biological factors anxiety symptoms (*p* = 0.001; odds ratio [OR], 23.13; 95% confidence interval [CI]: [2.87–186.10]) and pain (*p* = 0.001; OR, 1.61; 95% CI: 1.27–2.04). The associations between biological and environmental factors and a full night of sleep are shown in Table 2.

**Table 2:** Patients’ report of a full night of sleep and stressors

	Full night of sleep		*p* value	Odds ratio (95% CI)
	Yes	No		
**Biological factors**				
Anxiety symptoms	1 (1.4%)	19 (26.7%)	0.001	23.13 (2.87–186.10)
Pain	0	16 (22.5%)	0.001	1.61 (1.27–2.04)
**Environmental factors**				
Noise	6 (8.4%)	17 (23.9%)	0.121	2.60 (0.87–7.74)
ICU lights	6 (8.4%)	8 (11.2%)	1.00	0.90 (0.27–2.94)

Note: *p* values were obtained using the chi-squared test

A higher percentage of patients in the younger group were affected by biological factors; 26 patients (72.2%) in the ≤55-year-old group and 16 patients (45.7%) in the older age group were affected by biological factors (*p* = 0.031, OR, 0.32; 95% CI: 0.12–0.86). A higher percentage of patients in the younger group was also affected by environmental factors: 17 patients (47.2%) in the ≤55-year-old group and 13 patients (37.1%) in the > 55-year-old group (*p* = 0.474). The distributions of biological and environmental factors according to age groups and duration of hospital stay are shown in Table 3.

**Table 3:** Frequency of factors according to age and duration of hospital stay

Age				
Biological factors	≤55 years (*n* = 36)	> 55 years (*n* = 35)	*p* value	Odds ratio (95% CI)
Anxiety symptoms	14 (38.8%)	6 (17.1%)	0.064	0.32 (0.10–0.98)
Pain	11 (30.5%)	5 (14.2%)	0.155	0.37 (0.11–1.23)
**Environmental factors**				
Noise	16 (44.4%)	7 (20%)	0.042	0.31 (0.10–0.90)
Lights	11 (30.5%)	3 (8.5%)	0.035	0.21 (0.05–0.84)
**ICU stay length**				
**Biological factors**	≤5 days of (*n* = 38)	> 5 days (*n* = 33)	*p* value	Odds ratio (95% CI)
Anxiety symptoms	4 (10.5%)	16 (48.4%)	0.001	8.00 (2.31–27.66)
Pain	7 (18.4%)	9 (27.2%)	0.407	1.66 (0.54–5.10)
**Environmental factors**				
Noise	8 (21%)	15 (45.4%)	0.042	3.12 (1.10–8.82)
Lights	5 (13.1%)	9 (27.2%)	0.231	2.47 (0.73–8.32)

Notes: *p* values were obtained using the chi-squared test

### Noise level

The global noise level was 62.45 ± 3.7 dB. When analyzed according to shift, the noise levels were 62.5 ± 3.72 dB during the morning shift, 62.88 ± 3.41 dB during the afternoon shift, and 61.89 ± 3.89 dB during the night shift.

**Fig. 2:**
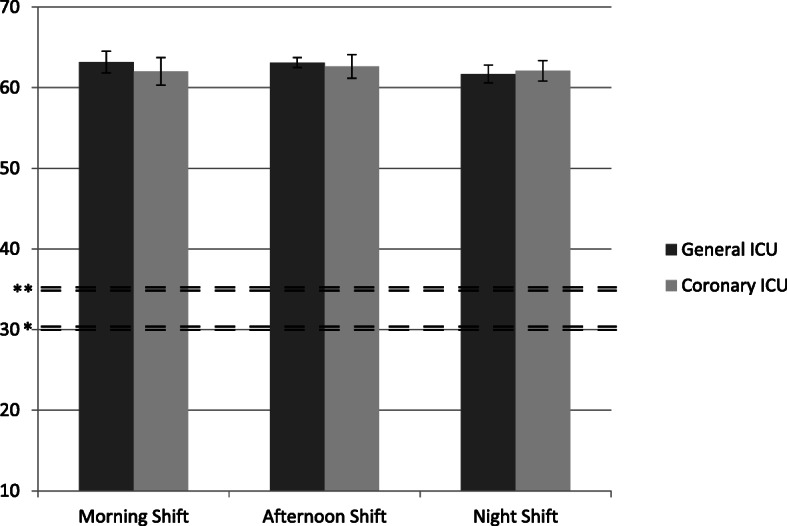
Comparison between the general ICU and the step-down unit sound levels shown according to work shift. * dB recommended by the World Health Organization (WHO) during the night. ** dB recommended by the WHO during the day

The global noise level was 62.66 ± 1.23 dB in the general ICU. The noise levels were 63.17 ± 1.33 dB, 63.11 ± 0.63 dB, and 61.69 ± 1.11 dB during the morning, afternoon, and night shifts, respectively. These differences were significant (*p* = 0.018, one-way ANOVA). The global noise level was 62.25 dB ± 1.45 in the step-down unit. The noise levels were 62.01 ± 1.71 dB, 62.65 ± 1.46 dB, and 62.08 ± 1.27 dB during the morning, afternoon, and night shifts, respectively (*p* = 0.654, one-way ANOVA). A comparison between noise levels in the general ICU and the step-down unit is shown in Fig. 2. The global and shift mean sound levels are shown in Table 4.

**Table 4:** Comparison of sound levels between different ICU units and shifts

Type of ICU	Global ICU stay	Morning shift	Afternoon shift	Night shift
**General ICU**				
1	62.95 ± 1.89	64.08 ± 2.99	62.66 ± 2.18	62.12 ± 1.10
2	64.63 ± 2.36	66.40 ± 3.25	64.00 ± 1.56	63.50 ± 2.26
3	60.41 ± 5.22	60.92 ± 5.24	60.92 ± 6.06	59.40 ± 4.40
4	63.63 ± 1.03	64.52 ± 1.97	64.20 ± 1.04	62.17 ± 0.88
5	59.56 ± 7.47	62.22 ± 1.79	58.54 ± 10.22	57.92 ± 10.91
6	61.66 ± 3.30	62.90 ± 1.63	61.70 ± 3.61	60.40 ± 2.59
7	63.20 ± 0.83	63.24 ± 1.49	64.02 ± 1.80	62.34 ± 1.15
8	60.80 ± 0.52	60.75 ± 0.64	61.40 ± 2.55	60.25 ± 3.46
**Step-down unit**				
9	61.56 ± 1.88	60.95 ± 3.28	61.60 ± 1.83	62.15 ± 0.79
10	61.56 ± 0.91	60.60 ± 1.21	62.48 ± 1.90	61.62 ± 1.02
11	63.43 ± 2.40	61.95 ± 1.57	62.48 ± 1.27	65.86 ± 6.42
12	63.86 ± 1.65	63.52 ± 2.55	64.00 ± 1.36	64.06 ± 2.19
13	65.15 ± 1.93	64.22 ± 1.06	65.48 ± 2.79	65.76 ± 3.17
14	62.02 ± 0.32	62.11 ± 0.56	62.23 ± 2.57	61.71 ± 2.06
15	61.16 ± 1.11	61.33 ± 2.29	61.65 ± 1.59	60.50 ± 1.40
16	60.62 ± 2.12	60.25 ± 2.94	60.95 ± 1.99	60.68 ± 3.32
**Total**	62.25 ± 2.93	62.33 ± 2.62	62.38 ± 3.60	62.03 ± 4.23

The data are expressed as mean dB ± standard deviation

**Fig. 3:**
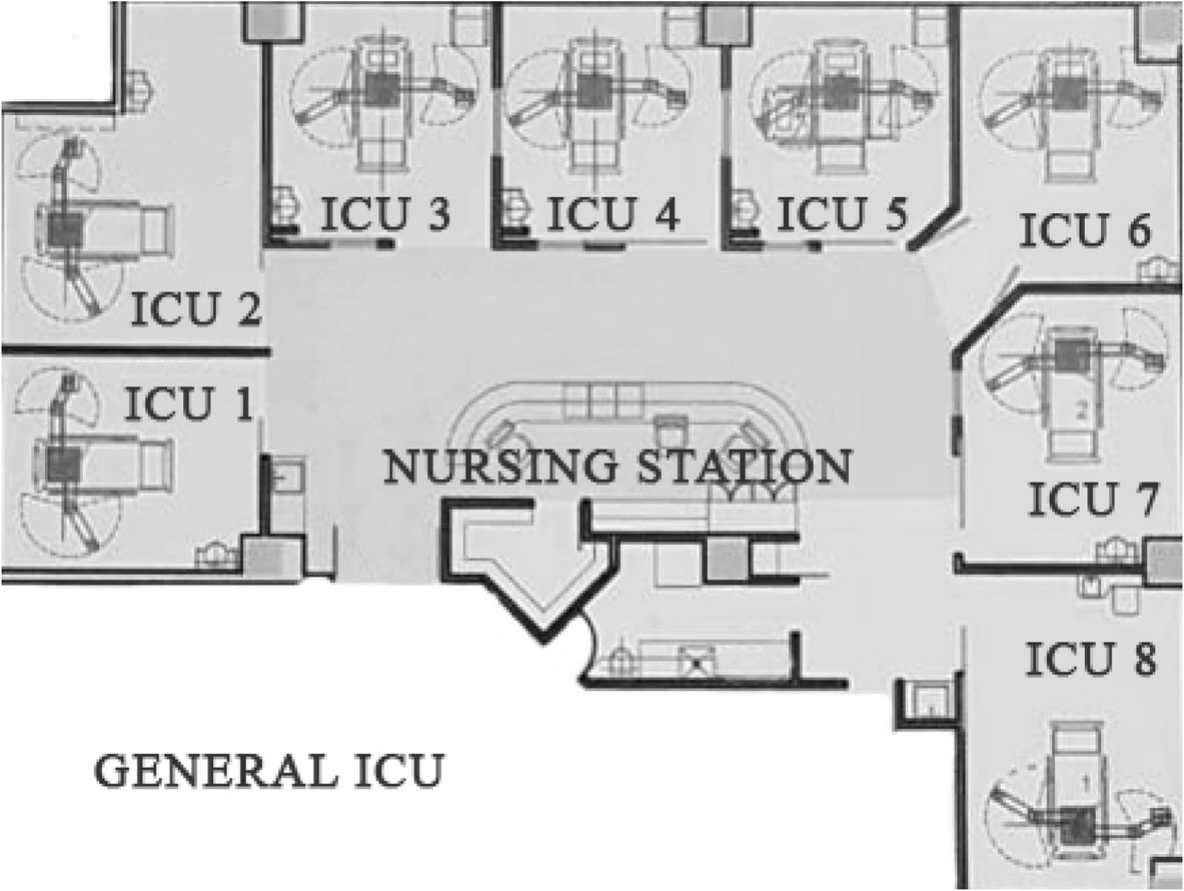
Structure of the general ICU

**Fig. 4:**
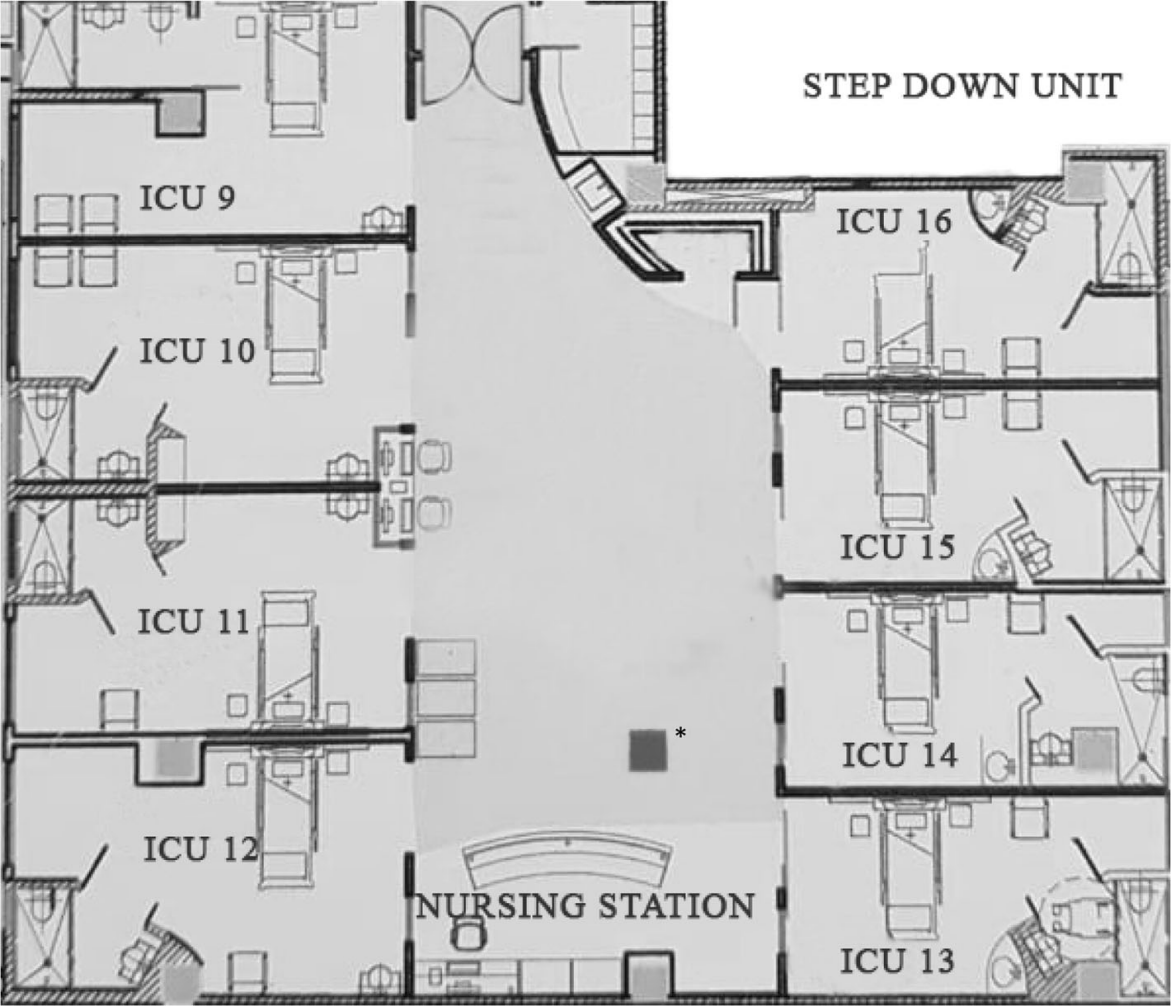
Structure of the step-down unit. *Structural beam

We examined whether the physical characteristics of the ICUs were related to the perception of sound. Schematic drawings of the layout of the units are shown in Figs. 3 and 4. The highest noise level was observed in unit 13 (65.15 ± 1.93 dB), followed by units 2 (64.63 ± 2.36 dB) and 12 (63.86 ± 1.65 dB). The lowest noise level was observed in units 5 (59.56 ± 7.47 dB), 3 (60.41 ± 5.22 dB), and 16 (60.62 ± 2.12 dB).

We examined whether the sound levels were associated with sleep disturbance caused by other stressors. The global noise level was significantly related to sleep disturbance in patients who reported pain as a biological factor during the night (*p* = 0.037). Similarly, the sound level during the night shift was significantly related to sleep disturbance in patients who reported pain as a biological factor during the night (*p* = 0.025). The associations between stressors and sound level are shown in Table 5.

**Table 5:** Associations between stressors and sound level

	Morning shift	*p* value	Afternoon shift	*p* value	Night shift	*p* value	Global ICU stay	*p* value
**Biological factors**								
Anxiety symptoms								
Present	61.81 ± 2.53	0.283	62.07 ± 2.72	0.597	61.75 ± 4.56	0.737	61.87 ± 2.76	0.488
Absent	62.55 ± 2.54		62.50 ± 3.09		62.15 ± 4.13		62.40 ± 3.00	
Pain								
Present	62.83 ± 1.67	0.269	62.98 ± 2.30	0.322	64.10 ± 4.49	0.025	63.30 ± 1.98	0.037
Absent	62.19 ± 2.83		62.20 ± 3.89		61.43 ± 3.99		61.94 ± 3.09	
**Environmental factors**								
Noise								
Present	62.22 ± 2.36	0.784	62.79 ± 2.00	0.411	62.62 ± 1.51	0.274	62.54 ± 1.49	0.459
Absent	62.40 ± 2.75		62.18 ± 4.15		61.75 ± 5.03		62.11 ± 3.41	
Lights								
Present	62.04 ± 2.82	0.661	62.10 ± 2.09	0.646	62.40 ± 1.46	0.537	62.18 ± 1.70	0.886
Absent	62.41 ± 2.58		62.45 ± 3.88		61.95 ± 4.67		62.27 ± 3.16	

The data are expressed as mean dB ± standard deviation, p values were obtained using Student’s t test

## Discussion

We found that environmental and biological factors negatively affected sleep quality in patients admitted to the ICU in a private hospital. These findings contrast with those of other studies that reported noise as the most prominent stressing factor [5, 21–23]⁠. Noise perceived by ICU patients is a major environmental factor that can interrupt patients’ sleep. We measured the sound levels at different times throughout the day and night and found that sound level ranged from 40 to 80 dB, as previously reported [13, 24–26]⁠. The sound levels measured in ICUs exceed those recommended by the WHO [16]⁠. The US Centers for Disease Control and Prevention (CDC) states that the maximum level of sound a human ear can sustain is 140–150 dB. Although patients are not exposed to extreme sound levels in the ICU, the CDC also states that 70–85 dB can be harmful after 2 h of exposure [27].⁠ This is vital information given that a study of the sound levels in ICU rooms and general ward rooms reported that ICU rooms were not only louder than those in general wards, but that the sound level appeared as several peaks > 85 dB across 24–72 h of recording in the ICU [20]⁠.

We found that the units with the most noise were located next to the entrance to the ICU and the nursing station in the step-down unit. Noise in the nursing station related mainly to chair movement, alarms, staff conversations, and telephones. An example of this difference was observed in unit 16, which had the lowest levels recorded from all other step-down units, probably because of the distance between the unit and the nursing station. Coincidentally, one unit (unit 14) was “blocked” from this sound source by a structural beam and its noise level was lower. For example, the mean sound levels were 65.15 dB in unit 13 and 63.86 dB in unit 12, but 62.02 dB in unit 14 (Fig. 4). This contrasts with the noise levels in unit 11 (63.43 dB), which had no structural beam blocking the sound (Fig. 4). A previous study showed that it is possible to reduce noise perception with architectonic modifications [22]⁠. Although the structural beam is part of the hospital’s construction and was not constructed to interrupt the noise, this observation suggests that structures within the ICU may help to reduce noise; this may be important because ICU units do not usually have doors or, if they do, they are kept partially open door to facilitate entrance by physicians and nursing personnel in case of emergency. However, this arrangement allows the noise of the ICU to enter the rooms and disturb the patients’ sleep, primarily during the night shift.

Second to noise, anxiety was the next most frequent biological stressor described by patients. Noise is considered to be one of the main aggravating factors related to the development of anxiety during a hospital stay [28]. We found that patients older than 55 years had a lower prevalence of anxiety during their hospital stay, suggesting that age may offer some level of protection against the development of anxiety symptoms. The presence of anxiety and stress symptoms seemed to be higher in younger patients but to be less disruptive with increasing length of hospital stay: a finding that has also been reported by Ayllón-Garrido et al. [29]⁠. It is important to identify anxiety and stress promptly during and after hospitalization in the ICU because patients experiencing these are susceptible to developing cognitive psychological distress, anxiety, and depression, which increase the risk of poor recovery and decrease quality of life after ICU discharge [30, 31].

The second most common biological stressing factor was pain. Studies have shown that 20–75% of patients report that pain alters their sleep [9, 29, 32, 33]⁠. Stress caused by noise has been shown to increase the pain threshold in both animal and human experimentation models [34, 35]⁠. In our patients, the presence of pain was significantly associated with perceived noise levels within the ICU for both the night shift and global hospital stay. These findings suggest that noise in the ICU during daytime can also affect patients’ sleep. It seems that the longer the hospital stay is, the stronger the effect of pain as a stressing factor that can affect patients’ sleep becomes. Additionally, we found that none of the patients who reported pain as a factor during the night had a full night of sleep during their hospital stay. Sleep deprivation can, to some extent, increase the stress of being hospitalized and decrease the patient’s pain threshold [36, 37].

Almost 20% of our patients associated their sleep disturbances with the ICU’s lighting. This is a higher percentage than that reported in the literature [38]⁠. ICUs require continuous lighting for management of patient care, but such lighting can suppress melatonin secretion, which can alter the circadian cycle [39]⁠. Even low lighting, such as indoor lighting, can affect a patient’s sleep schedule, although it is not a prominent factor that disrupts sleep [40–42]⁠. Some critically ill patients are placed in a decubitus position for > 24 h at a time, which can alter their circadian cycle and lead to modifications of their sleep schedule, for example by causing them to sleep during the day but remain awake at night because of the lack of natural lighting, loss of the sense of day and night, and excessive nocturnal noise. Alterations in the circadian cycle can affect the sleep cycle and thermal regulation, which may increase a patient’s susceptibility to complications such as sepsis and poor recovery in addition to their initial pathology [12, 41]⁠.

Physicians and hospital personnel should be aware that noise in the ICU can affect patients’ sleep patterns and that both environmental and biological factors can directly affect patients’ sleep. Studies have noted the importance of improving the environment to promote healthier sleep and overall quality of life during ICU admission [43–46]⁠. One proposed solution is the introduction of “quiet times” for patients admitted to the ICU, when patients are given a period of reduced noise and light stimuli with the objective of improving sleep. Several studies have reported improvement in sleep quality at night and in the overall ICU environment in both patients and hospital personnel after the implementation of quiet times [9, 47–49]⁠. These findings suggest that this intervention may help to prevent pain in patients rather than treating pain as it appears. Additionally, in patients already experiencing pain, pharmaceutic measures (such as NSAIDS, ketamine, or opioids) and nonpharmaceutical approaches (such as massage, hypnosis, ear plugs sleeping masks or music therapy) may be beneficial [2]. These options may also help relieve sleeping difficulties in different circumstances such as agitation, immobility, and sleep disturbances. Physicians can provide patients with medical and nonmedical means of controlling the stressing factors encountered in the ICU to maximize the therapeutic environment when treating an already complicated patient. Hopefully, this will lead to a faster and better recovery.

One limitation of this study is that sound was measured three times a day in the morning, afternoon, and night, because the research team could not perform 24 h continuous recording for the exact measurement of the sound levels in each unit. We compromised by measuring the sound level at different times each day to obtain a more realistic measurement of the global sound panorama in the ICU (e.g., visiting and nonvisiting hours, shift changes). Another limitation is the assessment of anxiety. We did not use a specific instrument to measure anxiety and relied on the patients’ subjective perception of anxiety.

## Conclusions

The most frequent factors associated with sleep disturbance in the ICU were anxiety symptoms, pain, ICU lighting, and noise made by hospital staff, vital sign monitors, and infusion pumps. The ability of a patient to recover in the ICU depends on good quality sleep, and this should be a priority for hospital personnel. We suggest the use of earplugs and sleeping masks for patients whose condition allows. We also encourage nursing and medical staff to decrease the noise made by their activities within the units, to be alert to medication that can disrupt patients’ sleep, and to devise strategies to adjust alarms to the individual needs of each patient. Implementation of these measures and other structural modifications may help reduce the sound levels and help patients in the ICU attain good sleep quality.

## Data Availability

The datasets used and/or analyzed during the current study are available from the corresponding author on reasonable request.

## Abbreviations

ANOVA: Analysis of variance
CDC: US Center for Disease Control and Prevention
CI: Confidence interval
dB: Decibel
ICU: Intensive care unit
OR: Odds ratio
RCSQ: Richards–Campbell Sleep Questionnaire
WHO: World Health Organization
